# The energetic barrier to single-file water flow through narrow channels

**DOI:** 10.1007/s12551-021-00875-w

**Published:** 2021-11-23

**Authors:** Juergen Pfeffermann, Nikolaus Goessweiner-Mohr, Peter Pohl

**Affiliations:** Institute of Biophysics, Johannes Kepler University Linz, Linz, Austria

**Keywords:** Confined geometry, Hydrogen bonding, Permeability, Activation energy, Transition state theory, Entropy and enthalpy

## Abstract

Various nanoscopic channels of roughly equal diameter and length facilitate single-file diffusion at vastly different rates. The underlying variance of the energetic barriers to transport is poorly understood. First, water partitioning into channels so narrow that individual molecules cannot overtake each other incurs an energetic penalty. Corresponding estimates vary widely depending on how the sacrifice of two out of four hydrogen bonds is accounted for. Second, entropy differences between luminal and bulk water may arise: additional degrees of freedom caused by dangling OH-bonds increase entropy. At the same time, long-range dipolar water interactions decrease entropy. Here, we dissect different contributions to Gibbs free energy of activation, Δ*G*^‡^, for single-file water transport through narrow channels by analyzing experimental results from water permeability measurements on both bare lipid bilayers and biological water channels that (i) consider unstirred layer effects and (ii) adequately count the channels in reconstitution experiments. First, the functional relationship between water permeabilities and Arrhenius activation energies indicates negligible differences between the entropies of intraluminal water and bulk water. Second, we calculate Δ*G*^‡^ from unitary water channel permeabilities using transition state theory. Plotting Δ*G*^‡^ as a function of the number of H-bond donating or accepting pore-lining residues results in a 0.1 kcal/mol contribution per residue. The resulting upper limit for partial water dehydration amounts to 2 kcal/mol. In the framework of biomimicry, our analysis provides valuable insights for the design of synthetic water channels. It thus may aid in the urgent endeavor towards combating global water scarcity.

## The challenge: Development of artificial water channels

The global water scarcity problem intermittently affects 2/3 of the world’s population and 1.1 billion people all year round. It calls for radically improved energy-saving water purification technologies (Werber et al. 2016). Nanofiltration membranes with pores that are both highly selective and conductive for water may be part of the solution (Barboiu 2012; Werber and Elimelech 2018; Song et al. 2018; Chowdhury et al. 2018). In case of carbon nanotubes (CNTs), in silico dissection of the tradeoff between flow rate and salt rejection suggests an optimum diameter of 1.1 nm (Thomas and Corry 2016).

Such explorations of hydrodynamics and related transport at the smallest scales would have been impossible without the fabrication of nanofluidic devices amenable to systematic investigations. The interested reader finds an illuminating outline of the developments in nanofluidics elsewhere (Bocquet 2020). We highlight one critical observation for our further considerations: the inapplicability of the no-slip condition at the molecular scale. Perfect slip at the intraluminal water-channel interface was already observed for the peptide channel gramicidin nearly five decades ago (Levitt et al. 1978; Rosenberg and Finkelstein 1978). Otherwise, water transport through channels with a lumen as wide as one water molecule would be impossible. With increasing diameter to tens of nanometers, the perfect slip gradually changes to an imperfect slip (for a review, see Lauga et al. 2007). As a result, narrow CNTs conduct water at rates far exceeding geometry-based predictions from Hagen-Poiseuille’s law (Corry 2008; Secchi et al. 2016a).

Nature offers solutions for water desalinization with perfect slip and perfect selectivity. Eventually, the design of artificial pores strives towards achieving these capabilities that biological systems have already acquired (Park et al. 2017). As a prerequisite, we have to understand the molecular mechanisms that evolution has spent a long time adapting. Many plasma membrane channels facilitate single-file transport: their conduction pathway is so narrow that water molecules and/or ions cannot overtake each other (Horner and Pohl 2018b). These single-file transporters have distinct selectivities: potassium- and sodium-selective ion channels are involved in neural transmission while aquaporins are dedicated proteinaceous water conductors. An analysis of their water-transporting capability (Pohl et al. 2001; Saparov and Pohl 2004; Hoomann et al. 2013) as a function of pore structure is bound to reveal essential lessons for the design of artificial water channels (Baaden et al. 2018b, 2018a).

## Aims

In this contribution, we aim at enforcing the view that (i) channel structure determines the energetic barrier to water flux and (ii) the barrier height governs the resulting water flux. We limit the analysis to that of pores facilitating single-file water transport. Recent reports of artificial channels with a combination of supposedly high single-channel permeability, *p*_f_, and high Arrhenius activation energy, *E*_A_, prompted the effort (see §4). The analysis of experimental data presented in §6 shows that *E*_A_ is the principal constituent of Gibbs free energy of activation, Δ*G*^‡^, for single-file water transport through narrow channels.We show that no increment in the entropy of intraluminal water compensates for the reported *E*_A_ values (see §6). Accordingly, *E*_A_ is an excellent tool for predicting the maximum *p*_f_ value. Importantly, water partitioning into the channel may not serve as an explanation for high *E*_A_ values. Partitioning expenses are limited to 2 kcal/mol. In contrast, high *E*_A_ values indicate a water pathway distinct from facilitated diffusion through channels (see §4).

## Design principles for selectivity in biological water channels

**Fig. 1:**
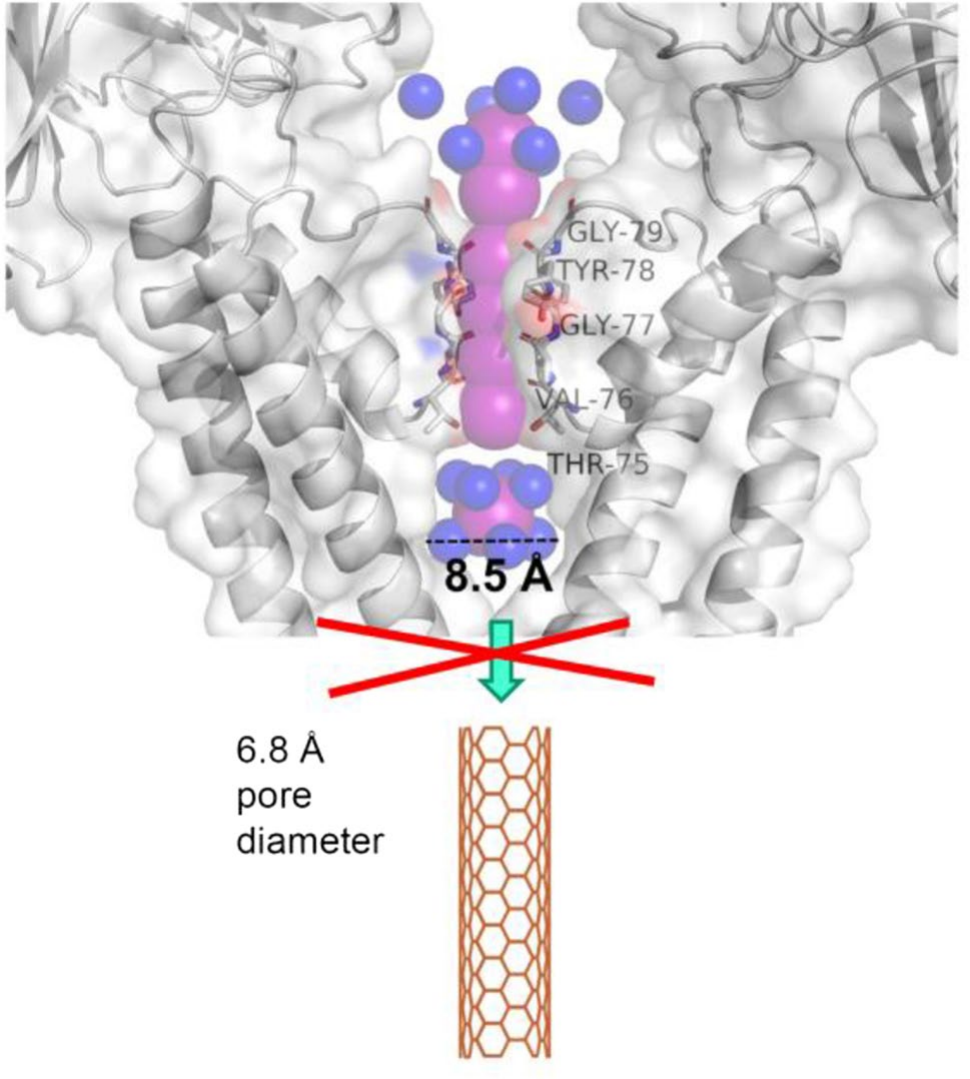
Hydrated cations are too large to enter narrow single-walled CNTs. The upper part shows the crystal structure of the selectivity filter of the bacterial potassium channel KcsA and part of its cavity (PDB: 1K4C) (Zhou et al. 2001). Both the front and back KcsA subunits are cut away for clarity. Backbone carbonyl oxygens of the indicated amino acids act as surrogates for the waters of K^+^ hydration. K^+^ and water are shown as pink and blue spheres, respectively. The lowest K^+^ localized in the aqueous cavity of the channel is depicted with its hydration shell. The diameter of the hydrated ion exceeds the inner diameter of the CNT depicted at the bottom

Size exclusion represents the primary selectivity mechanism of most channels. However, excluding anything physically larger than a molecule of water represents an insufficient design principle for selective pores (Song and Kumar 2019; Song et al. 2020; Epsztein et al. 2020). Reducing pore width to the diameter of a single water molecule does not necessarily preclude ion flow. For example, potassium-selective ion channels offer surrogates for the waters of hydration, thereby enabling the passage of dehydrated potassium ions (K^+^) through a narrow selectivity filter (Zhou et al. 2001). In this selectivity-determining region, carbonyl oxygens of the polypeptide backbone are arranged around the K^+^ binding sites in a geometry that closely resembles the position of water oxygens in the ion’s first hydration shell (Dutzler et al. 2002) (Fig. 1). In the absence of such surrogates for the waters of hydration, ion permeation is energetically highly unfavorable (Noskov and Roux 2007)—ripping K^+^ of its entire hydration shell costs about 80 kcal/mol (Friedman and Krishnan 1973).

Water-selective channels exploit this feature. While the conduction pathway of aquaporins is also roughly one water molecule wide, it lacks surrogates for the waters of ion hydration (Murata et al. 2000), and thus, the passage of small cations and anions is blocked (Saparov et al. 2001, 2005; Tsunoda et al. 2004). The aquaporin pore is also equipped with positive charges to prevent protons from permeating the channel (de Groot and Grubmuller 2001; Tajkhorshid et al. 2002). One positive charge is placed in a constriction area and partial positive charges are provided by two halve helices that point with their positive ends into the axial center of the pore. As a result, aquaporins offer an excellent selectivity: less than one ion is transported per 10^9^ water molecules (Pohl et al. 2001). Removal of positive charges from both cationic filters by point mutations renders aquaporins proton-conductive (Wu et al. 2009).

## Single-file water transport and selectivity in artificial channels

Narrow CNTs do not offer surrogates for the waters of ion hydration, thereby excluding ions analogously to aquaporins. The water-transporting characteristics of such CNTs have been numerously assessed in silico (Hummer et al. 2001; Joseph and Aluru 2008; Zuo et al. 2010; Zhang et al. 2013). Experimental studies on membranes comprising somewhat larger CNTs (inner diameter (ID) about 1–2 nm) (Holt et al. 2006) and individual tubes (ID 10–50 nm) (Secchi et al. 2016b) largely agree with the computational studies, while only recently emerging experimental results on water transport through sub-8-Å CNTs are debated (see below).

Molecular dynamics (MD) simulations suggest that ion exclusion by nanopores depends on ionic species (e.g., concerning coordination number, dehydration energy, and crystal radius especially for exceedingly narrow pores), interactions between ions and the pore walls (Song and Corry 2009; Secchi et al. 2016a), the structure of the hydration shell within the channel interior (Li et al. 2015), and applied pressure (Thomas and Corry 2016). Detailed cost calculations have been performed for NaCl and KCl in CNTs with IDs between about 3 and 9 Å at applied pressures of around 100–200 MPa (in comparison, the osmotic pressure of seawater is around 3 MPa); these resulted in a penalty of ≈23 kcal/mol for K^+^ entering (6,6) CNTs with an ID of 4.7 Å (Song and Corry 2009). In these narrow tubes, K^+^ maintained contact with two neighboring waters—one preceding and one following the ion—during the passage; i.e., desolvation was only partial. Under in vitro conditions, such a high barrier would render the tubes practically impermeable to K^+^. Nevertheless, we note that the ≈23 kcal/mol likely underestimates the actual costs. Observations from the same MD calculations corroborate this conclusion: the costs for complete K^+^ dehydration were determined to be only 53 kcal/mol (Song and Corry 2009)—yet the experimentally determined value amounts to about 80 kcal/mol (Friedman and Krishnan 1973). Recent MD simulations equally confirmed ion exclusion by (6,6) CNTs (Su et al. 2019).

In sub-2-nm-diameter CNTs where the energetic costs for removing hydrating water molecules from ions upon entering are smaller due to reduced steric constraints, charged moieties at the pore ends can confer some selectivity (Fornasiero et al. 2008). For example, carboxylate-modified CNTs of roughly 1.6-nm diameter require the application of membrane potential for K^+^ to enter (Choi et al. 2011). However, static charges attract counterions: e.g., while CNT functionalization with carboxyl groups rejects chloride, sodium rejection is decreased (Thomas and Corry 2016)—indeed, this strategy might be employed by cation channels with exposed negative charges in a vestibule near the selectivity filter to increase cation conductance (Latorre et al. 2017). Eventually, embedding CNTs bearing zwitterionic groups into polyamide membranes increased ion rejection by combined electrostatic and steric effects (Chan et al. 2013).

Surprisingly, there are experimental reports of K^+^ and chloride ion permeation through CNTs with an outer diameter (OD) of 8 Å embedded in lipid bilayers (Tunuguntla et al. 2017). However, the crystal structure of a K^+^ with a complete hydration shell—captured as part of the high-resolution structure of a bacterial K^+^ channel (Zhou et al. 2001) — has a diameter of roughly 8.5 Å. Thus, the diameter of hydrated K^+^ is too large to fit into these CNTs with an ID of only 6.8 Å (Fig. 1). Complete or partial removal of the hydration shell is improbable due to the associated high energetic penalties stated above. Thus, ion passage at the interface between CNT and lipid bilayer provides a plausible explanation for the experimental observation (Horner and Pohl 2018a). Notably, the debate whether ions took an extraluminal pathway could be settled by inhibition experiments in which physical occlusion of the bilayer-embedded CNTs would have to block ion passage.

Furthermore, such inhibition experiments would clarify the pathway water takes in the case of bilayer-embedded CNTs. The initially published Arrhenius activation energy, *E*_A_, for water transport of up to 25 kcal/mol (Tunuguntla et al. 2017) suggests that most, if not all, water molecules pass through the bilayer or traverse the CNT-bilayer interface (Horner and Pohl 2018a). The observed pH-dependence of *E*_A_ corroborates this interpretation, as CNT partitioning is equally pH-dependent (Tunuguntla et al. 2017). Furnished with carboxyl groups at their ends, tube partitioning into the lipid bilayer is facilitated at low pH values since protonation of these groups renders them electrically neutral. A subsequent study reported a lower *E*_A_ of only 5.3 kcal/mol (Li et al. 2020). Since it neither investigated the pH-dependence of water flow nor cation permeation, their transport pathways remain elusive.

Interestingly, a variety of artificial water channels have been reported to have high unitary water permeabilities, *p*_f_, and, at the same time, high *E*_A_ values for water transport. Besides narrow sub-8-Å CNTs (Tunuguntla et al. 2017; Li et al. 2020), aquafoldamers (Shen et al. 2020; Roy et al. 2021) provide an additional example. At first glance, the combination of high *p*_f_ and high *E*_A_ appears odd because a steeply uphill pathway is seldom a fast one. Biological channels facilitate water flow by lowering this barrier, i.e., by reducing *E*_A_. Figuratively speaking, biological channels represent a tunnel that shortens the arduous path over the mountain. How can an artificial canal offer the same transport efficiency as a biological one if the path it provides still leads over the top of the mountain?

## On the entropy of intraluminal waters

A theoretical possibility is that a sufficiently large entropy gain offsets the enthalpy expenditure:

1 $$\Delta {G}^{\ddagger }={\Delta {H}^{\ddagger }-T\Delta {S}^{\ddagger }= E}_{{\rm A}}-RT-T\Delta {S}^{\ddagger }$$

where $\Delta {G}^{\ddagger }$, $\Delta {H}^{\ddagger }$, and $\Delta {S}^{\ddagger }$ are the Gibbs free energy, the enthalpy, and the entropy of activation, respectively, while *R* and *T* denote the molar gas constant and absolute temperature. That is, even though *E*_A_ for some artificial water channels is comparable to or even higher than *E*_A_ for the lipid matrix, $\Delta {G}^{\ddagger }$ could still, theoretically, be small.

Such a scenario is supported by the in silico observation of an entropy-stabilized, vapor-like water phase in small CNTs (Pascal et al. 2011). However, the entropy gain of roughly 3 kcal/mol reported there turned into a small loss of ≈0.8 kcal/mol in a later MD simulation (Waghe et al. 2012). Infrared spectroscopy advocates an increase in entropy of intraluminal waters by observing “free”/dangling OH-bonds facing the nanotube wall (Dalla Bernardina et al. 2016). Yet simulations show that only the initial water loading of the tubes is dominated by entropic (both translational and rotational) components (Garate et al. 2014). In the filled state, dipolar interactions dominate (Kofinger et al. 2008). Dipolar ordering of water has also been observed in aquaporins (de Groot and Grubmuller 2001; Tajkhorshid et al. 2002). Such order is bound to decrease water entropy in addition to the confinement into a one-dimensional water wire. Consequently, it appears questionable whether such pronounced increments in entropy, required for compensating the reported high *E*_A_ values, are attainable.

## Unitary water permeability and activation energy are intricately linked

To clarify whether changes in water entropy make a noticeable contribution to the energetics of permeating water molecules, we analyze data on *E*_A_ and water permeability for biological channels and bare lipid bilayers. The analysis is based on our previously published application of transition state theory (Horner and Pohl 2018a). First, we introduce the “hopping rate,” *r*_h_, with which the water chain moves forward or backward (Berezhkovskii and Hummer 2002):

2 $${r}_{{\rm h}}={p}_{{\rm f}}{/v}_{{\rm w}}$$

where *v*_w_ = 3 × 10^−23^ cm^3^ is the volume of one water molecule. Second, we use the universal transition state theory to link *r*_h_ and Δ*G*^‡^:

3 $${r}_{\rm{h}}={\nu }_{0}\cdot {\rm exp}(-\Delta {G}^{\ddagger }/{R}T)$$

where *ν*_0_ is the universal attempt frequency, *ν*_0_ = *k*_B_∙*T*/*h* ≈ 6.2 × 10^12^ s^−1^ at room temperature (*k*_B_ is Boltzmann’s and *h* is Planck’s constant). From Eqs. (2) and (3), we find (Horner and Pohl 2018a):

4 $${p}_{\rm{f}}={\nu }_{0}{v}_{\rm{w}}\cdot {\rm exp}(-\Delta {G}^{\ddagger }/{R}T)$$

**Fig. 2:**
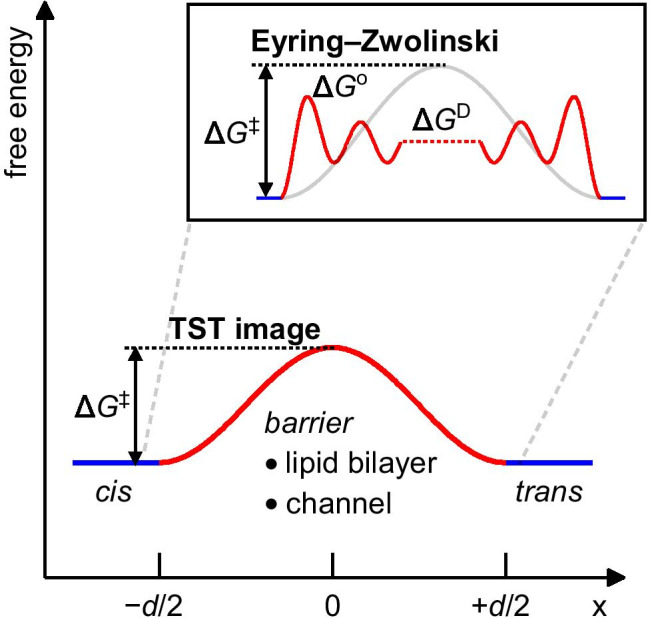
Translocation of water across the bare lipid bilayer or a water flow-facilitating channel necessitates surpassing energetic barriers, which are summarized in Δ*G*^‡^. According to the theory of absolute reaction rates (transition state theory, TST), Δ*G*^‡^ reports on the rate of a reaction—here, the translocation of water from one (*cis*) to the other (*trans*) side of the membrane, be it by traversing the hydrophobic core of the lipid bilayer or a proteinaceous pore lined with polar groups. The inset provides a more detailed picture of the process of water permeation, inspired by Zwolinski et al. (1949)*.* The authors envisioned the rate of translocation of water through the lipid bilayer to be determined by (a) partitioning into and out of the bilayer, described by the distribution coefficient exp(−Δ*G*^o^/*RT*), and (b) diffusive movement within the hydrophobic interior as hopping between local minima with a rate proportional to exp(−Δ*G*^D^/*RT*). Qualitatively, water permeation in a single file through a biological channel may be described similarly. Upon entering, water effectively loses H-bonding partners, and the advancement of the file requires breaking H-bonds between intraluminal waters and the pore lumen.

Equation (4) describes water translocation in terms of a one-step reaction, reflecting the collective motion of water molecules in the single file (Fig. 2). Water molecules on the two sides of the membrane may be regarded as reactants and products. The molecules in the channel are in the activated state, characterized by $\Delta {G}^{\ddagger }$. Expression (4) does not build on pore-specific assumptions. Accordingly, it should also work for simple water diffusion across lipid bilayers. A few simple transformations show that this is indeed the case. First, we divide both sides of Eq. (4) by the cross section of a water molecule, *A* = π *r*^2^, where *r* = 1.5 Å:

5 $${P}_{{\rm f}}=\frac{{4\nu }_{0}r}{3}\cdot {\rm exp}(-\Delta {G}^{\ddagger }/{R}T)$$

where *P*_f_ (in cm/s) denotes an effective osmotic permeability. The derivation of Eq. (5) used the equality 4∙*r*/3 = *v*_w_/*A*. Following Eyring-Zwolinski’s lead (Zwolinski et al. 1949), we then recognize the improbability of the water molecule to cross the hydrophobic part of the bilayer of thickness *d* (= channel length) in a single jump (Fig. 2). Defining jump length *λ*, where *λ* < *d*, by Eq. (6):

6 $${\lambda }^{2}=\frac{4r\cdot d}{3}$$

brings us to Eq. (7):

7 $${P}_{{\rm f}}=\frac{{{\lambda }^{2}\nu }_{0}}{d}\cdot {\rm exp}\left(-\Delta {G}^{\ddagger }/{R}T\right)$$

Equation (7) is the Eyring-Zwolinski equation. Its original form describes the permeation of uncharged molecules across lipid bilayers (Hannesschlaeger et al. 2019). $\Delta {G}^{\ddagger }$ contains the energetic terms for partitioning $\Delta {G}^{{\rm o}}$ into the membrane (or channel) as well as diffusion $\Delta {G}^{{\rm D}}$ across channel or membrane (Fig. 2).

In principle, both *λ* and *d* should depend on the structure of the membrane or channel. We avoid this ambiguity by using Eq. (5). For an analysis of entropic and enthalpic contributions to water translocation, we rewrite Eq. (5) with the help of Eq. (1):

8 $${P}_{{\rm f}}=\frac{{4r\nu }_{0}}{3}\cdot {\rm exp}\left(-\frac{{E}_{\rm A}}{{R}T}\right)\cdot {\rm exp}\left(1+\frac{\Delta {S}^{\ddagger }}{{R}}\right)$$

**Fig. 3:**
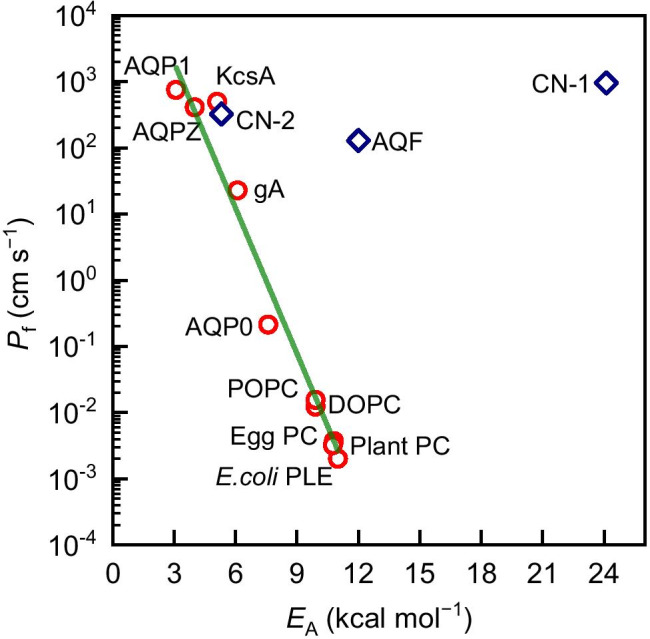
The effective water permeability, *P*_f_, of lipid bilayers and single-membrane channels as a function of the Arrhenius activation energy, *E*_A_, in a semilogarithmic representation. The plot uses the following transformation: *P*_f_ = *p*_f_/*A*, where *p*_f_ is the unitary channel permeability, and *A* denotes the cross-sectional area of one water molecule. The green line is a linear fit of Eq. (8) to the data. Accordingly, the slope is equal to −1/*RT*ln(10). Taking *v*_0_ = 6.2 × 10^12^ s^−1^ allows calculating $\Delta {S}^{\ddagger }$ ≈ –0.1∙*R*. Thus, the plot indicates a negligible entropy of activation for water diffusion across lipid bilayers and membrane channels. Below, we list the sources of the plotted data next to the abbreviations used in the figure: aquaporin-1, AQP1 (Horner et al. 2015; Zeidel et al. 1992); aquaporin-Z, AQPZ (Horner et al. 2015; Pohl et al. 2001); bacterial potassium channel, KcsA (Horner et al. 2015; Saparov and Pohl 2004); gramicidin A, gA (Pohl and Saparov 2000; Boehler et al. 1978); aquaporin-0, AQP0 (Zampighi et al. 1995; Kumar et al. 2012); palmitoyl oleoyl phosphatidylcholine, POPC (Huster et al. 1997); dioleoyl phosphatidylcholine, DOPC (Huster et al. 1997); polar lipid extract from *Escherichia coli*, *E. coli* PLE (Saparov and Pohl 2004; Pluhackova and Horner 2021); egg and plant phosphatidylcholine, Egg PC and Plant PC (Fettiplace and Haydon 1980); CNT, CN-1 (Tunuguntla et al. 2017); CNT, CN-2 (Li et al. 2020); aquafoldamer, AQF (Shen et al. 2020).

A semilogarithmic plot of *P*_f_ as a function of *E*_A_ for various channels and lipids reveals a linear function with the slope −1/*RT*ln(10) (Fig. 3). Inserting *v*_0_ = 6.2 × 10^12^ s^−1^ allows determining $\Delta {S}^{\ddagger }$ ≈ −0.87 J/(mol∙K) from the intercept, i.e., $\Delta {S}^{\ddagger }\ll -{R}$. We conclude that the entropic contributions to $\Delta {G}^{\ddagger }$ are negligibly small. Moreover, the small value of $\Delta {S}^{\ddagger }$ explains the excellent agreement between the theoretical prediction of Eq. (4) and carefully determined experimental *p*_f_ values when approximating $\Delta {G}^{\ddagger }$ by experimentally determined ${E}_{{\rm A}}$ values (Horner and Pohl 2018a, b). We refer to experimental data obtained by (i) proper accounting for unstirred layer effects and (ii) relying on an accurate count of the reconstituted channels per area. To that effect, a review of practical experimental approaches and potential pitfalls has been published recently (Horner and Pohl 2018b). Instead of presenting individual ${E}_{{\rm A}}$ measurements—each performed by quantifying water permeability as a function of temperature—we merely cite the results here (Fig. 3).

Observing Fig. 3, one notes that recently reported ${E}_{{\rm A}}$ values for artificial water channels (CN-1 and AQF) are too high to agree with the observed water transport rates. For the CNTs with ${E}_{{\rm A}}$ = 24.1 kcal/mol discussed in §4 (CN-1 in Fig. (3)), the corresponding ${P}_{{\rm f}}$ value amounts to 962 cm/s. Aquafoldamers (Shen et al. 2020) have a lipid-like ${E}_{{\rm A}}$ of 12 kcal/mol but a ${P}_{{\rm f}}$ of 130 cm/s, which is five orders of magnitude higher than that typical of lipids. Since the change in entropy is negligible, i.e., $T\Delta {S}^{\ddagger }\ll -{R}T$ (Fig. 3), no sufficient compensation for the extreme expenses in ${E}_{{\rm A}}$ is available. In other words, entropy does not serve to decrease $\Delta {G}^{\ddagger }$. Thus, Fig. 3 suggests that neither the discussed CNTs nor the aquafoldamers in question facilitate water transport at the reported rates.

## The role of hydrogen bonds

**Fig. 4:**
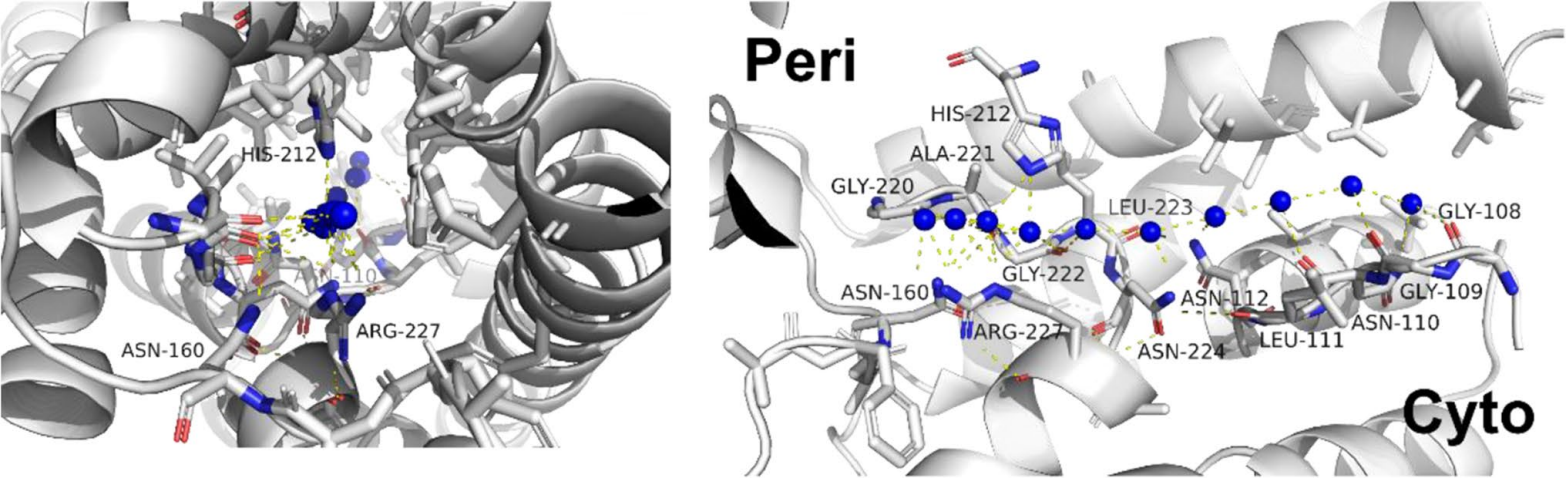
Water molecules (blue spheres) in the single-file configuration as captured by the crystal structure of the yeast aquaporin (PDB: 3ZOJ) (Kosinska Eriksson et al. 2013). The left panel affords a view along the pore axis. The right panel allows a lateral view from within the membrane: the water wire spans from the periplasmic side (left) to the cytoplasmic side (right). Residues that are H-bonding with intraluminal waters are labeled

Since the number of hydrogen bond-forming residues provided by the pore lumen, *N*_H_, is the primary determinant of ${p}_{{\rm f}}$ in a variety of biological single-file water-conducting channels (Horner et al. 2015), we may analyze the energetic impact of a single H-bond between water and the pore wall on single-file water transport. Therefore, we calculated $\Delta {G}^{\ddagger }$ from ${p}_{{\rm f}}$ with the help of Eq. (4). The water-containing yeast aquaporin structure—captured at sub-Angstrom resolution—illustrates H-bond formation within nanopores (PDB: 3ZOJ) (Kosinska Eriksson et al. 2013). It shows a total of *N*_H_ = 13 H-bond accepting or donating pore-lining residues relevant to the energetic balance of water permeation (Fig. 4).

**Fig. 5:**
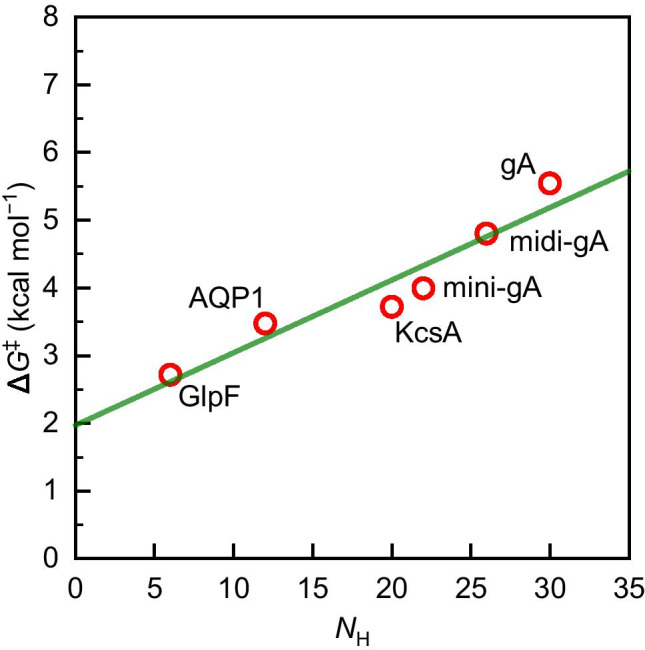
The Gibbs free energy of activation, $\Delta {G}^{\ddagger }$, calculated from *p*_f_ according to Eq. (4), plotted as a function of the number of hydrogen bonding residues, *N*_H_, offered by the channel walls. The linear regression (green line) suggests 2.0 kcal/mol expense for facilitated water transport independent of intraluminal H-bonding and 0.1 kcal/mol per H-bonding pore-lining residue

The involved residues are conserved in orthodox (water-selective) aquaporins, i.e., the human aquaporin-1, AQP1, and the bacterial aquaporin-Z, AQPZ. AQPZ has a lower *p*_f_ than AQP1 (Fig. 3), because it switches between conducting and non-conducting conformations (Jiang et al. 2006). The same holds for human AQP0 (Gonen et al. 2004), which appears to spend more time in its water-impermeable conformation than AQPZ. The bacterial channel GlpF belongs to the family of aquaglyceroporins. It has a somewhat shorter single-file region (Jensen and Mouritsen 2006; Hashido et al. 2007). With *N*_H_ = 6, it is the aquaporin with the highest known *p*_f_. In our analysis of $\Delta {G}^{\ddagger }$ as a function of *N*_H_ (Fig. 5), we include only constitutively open channels since energetic expenses for protein gating would confound the correlation. We note that the observed dependence of *p*_f_ on *N*_H_ may be understood qualitatively considering the effect of hydrogen bonding on the diffusion of water in *n*-alkanes versus *n-*alcohols (Su et al. 2010). The diffusion of water molecules in *n-*alcohols is delayed by relatively longer residence times compared to the non-H-bonding *n-*alkanes.

We find that $\Delta {G}^{\ddagger }$ is a linear function of *N*_H_ (Fig. 5). The slope indicates that every H-bond donating or receiving pore-lining residue contributes an increment ${\Delta \Delta }{G}^{\ddagger }$ of 0.1 kcal/mol to $\Delta {G}^{\ddagger }$. At first glance, this ${\Delta \Delta }{G}^{\ddagger }$ appears small compared to the energy of an H-bond. Yet, it becomes reasonable upon accounting for the collective motion of a water wire whose length matches the hydrophobic thickness of the bilayer. The Gedankenexperiment envisions a channel formed from bulk water molecules that accommodates a wire of 8 water molecules (the length of the single-file region in aquaporins). Only two out of four hydrogen bonds would break per wire molecule while moving through the channel since the hydrogen bonds between the wire molecules remain intact; i.e., the advancement occurs collectively. Thus, *N*_H_ is equal to 16 for such a pore. Channeling water molecules through such a pore would evoke a contribution from hydrogen bonds: ${N}_{{\rm H}}\times {\Delta \Delta }{G}^{\ddagger }$ = 1.6 kcal/mol according to Fig. 5. Adding the energetic contributions apart from those by intraluminal H-bonds with the channel walls, which we find from the intercept of the linear model in Fig. 5 to be $\Delta {G}^{\ddagger }\left({N}_{{\rm H}}=0\right)$ = ${\Delta \Delta }{G}_{{i}}^{\ddagger }=$ 2 kcal/mol, brings the total expense in free energy to $\Delta {G}^{\ddagger }$ = 3.6 kcal/mol. Inserting this value into Eq. (1) and taking into account $T\Delta {S}^{\ddagger }$ ≈ 0 (Fig. 3) allows calculating ${E}_{{\rm A}}$ = 4.2 kcal/mol. This is very close to the value of 4.6 kcal/mol found for water self-diffusion at 25 °C (Wang et al. 1953). Thus, our analysis is in line with the commonly accepted energetics of water diffusion.

## The penalty for partial water dehydration upon entering the single file

We conceive two different processes which may have contributed to ${\Delta \Delta }{G}_{{i}}^{\ddagger }$ (the energetic contribution to $\Delta {G}^{\ddagger }$ independent of intraluminal H-bond formation to the channel walls). First, there is an expense for “normal” diffusion that adds to the expense for breaking and reforming hydrogen bonds. The activation barrier for “normal” diffusion amounts to about 1.6 kcal/mol in bulk water (Gillen et al. 1972). Yet, intraluminal water does not necessarily face the same barrier. Conceivably, it is lower in wider pores and higher in narrower pores. Aquaporin-1 provides an example for the effect of pore geometry: according to MD simulations, constriction sites—as represented by the NPA-motif and ar/R region—pose sizable barriers (de Groot and Grubmuller 2001).

Second, there is a penalty $\Delta {G}^{{\rm o}}$ for entering the single file, mainly due to partial water dehydration at the channel mouth. If intraluminal H-bond donating or receiving residues affected this penalty, it would already be accounted for by ${\Delta \Delta }{G}^{\ddagger }$. Following Eyring-Zwolinski (Zwolinski et al. 1949) and assuming that (i) the activation barriers for diffusion, $\Delta {G}^{{\rm D}}$ (comprising both normal and H-bond-related diffusion), and $\Delta {G}^{{\rm o}}$ are the predominant contributors to $\Delta {G}^{\ddagger }$, and (ii) these contributions are additive as suggested by Fig. 5:

9 $$\Delta {G}^{\ddagger }\approx\Delta {G}^{{\rm D}}+\Delta {G}^{{\rm o}}$$

we may rewrite Eq. (4):

10 $${p}_{{\rm f}}\approx {\nu }_{0}{v}_{{\rm w}}\cdot {\rm exp}\left(-\frac{\Delta {G}^{{\rm D}}}{{R}T}\right)\cdot {\rm exp}\left(-\frac{\Delta {G}^{{\rm o}}}{{R}T}\right)$$

Equation (10) distinguishes three steps: (i) partitioning of water into the channel, (ii) diffusion within the channel, and (iii) water exiting into the bulk (Fig. 2). The aforementioned steps co-occur for a collectively moving water file (Berezhkovskii and Hummer 2002; Bert et al. 2002; Zhu et al. 2004; Lynch et al. 2020). *p*_f_ (or ${P}_{\rm f}$) comprises the contributions of all three steps. The same holds for $\Delta {G}^{\ddagger }$ since we calculated it from *p*_f_.

$\Delta {G}^{\ddagger }$ reaches its minimum for *N*_H_ = 0, $\Delta {G}^{\ddagger }\left({N}_{{\rm H}}=0\right)$ = ${\Delta \Delta }{G}_{{i}}^{\ddagger }$, because $\Delta {G}^{{\rm D}}$ is minimal at this point (Fig. 5)—representing only the activation energy for “normal” diffusion. At ${N}_{{\rm H}}=0$, $\Delta {G}^{\ddagger }$’s remaining part is equal to $\Delta {G}^{{\rm o}}\approx {\Delta \Delta }{G}_{{i}}^{\ddagger }-\Delta {G}^{{\rm D}}\left({N}_{{\rm H}}=0\right)$. Assuming $\Delta {G}^{{\rm o}}$ > 0 (since $T\Delta {S}^{\ddagger }$ is negligible) and $\Delta {G}^{{\rm D}}$ > 0, ${\Delta \Delta }{G}_{{i}}^{\ddagger }$ poses an upper limit to $\Delta {G}^{{\rm o}}$. With ${\Delta \Delta }{G}_{{i}}^{\ddagger }$ = 2 kcal/mol, we have identified a useful estimate for $\Delta {G}^{{\rm o}}$.

The highly collective motion of water molecules effectively lowers the activation energy (de Groot and Grubmuller 2001). However, it would be erroneous to believe that the gain of two hydrogen bonds upon water exit compensates for the simultaneously occurring loss of two hydrogen bonds experienced by the incoming water molecule. The activation energy may still be quite impressive, as the work on K^+^ channels illustrates. A complex consisting of one K^+^ ion and one water molecule cannot diffuse through the selectivity filter of K^+^ channels if some surrogates for the waters of hydration flip out of the lumen (Cuello et al. 2010). As a result, an inactivated channel is impermeable for K^+^ (Cha and Bezanilla 1997). An inactivated channel conducts only water (Hoomann et al. 2013). It does not conduct H_2_O–K^+^ pairs, although the dehydration costs at the entry would be compensated by hydration at the exit.

Similarly, inferring the energetic barrier for a water molecule partitioning into the single-file region from the number of sacrificed H-bonds represents an oversimplification. The two broken hydrogen bonds would contribute ≈5 kcal/mol each, arriving at $\Delta {G}^{{\rm o}}$ ≈ 10 kcal/mol. According to this model, however, complete dehydration, i.e., removing all four neighbors, should cost ≈20 kcal/mol. In contrast, water vaporization costs only 10.5 kcal/mol (Price and Thompson 1969). MD simulations for gramicidin channels found $\Delta {G}^{{\rm o}}$ ≈ 2 kcal/mol (Portella et al. 2007) which is very much in line with our current analysis. Yet, it is worth noting that the same simulations underestimated $\Delta {G}^{{\rm D}}$.

The question arises whether $\Delta {G}^{{\rm o}}$ may be tuned to maximize water flux. Strategically placed charges may cause such variances since the costs for dehydrating cations and anions differ by a large margin. While positively charged amino acid side chains (derivatives of ammonium) are weakly hydrated, their negatively charged counterparts (carboxylates) bind water more strongly (Collins 1997; Kiriukhin and Collins 2002). Accordingly, the threefold higher water permeability of aquaporin-1 compared to aquaporin-4 has been attributed to the larger number of positive charges at the channel mouth in the former case (Horner et al. 2018). The number of hydrogen bond donating and receiving residues in the walls of both pores is identical. The difference in $\Delta {G}^{\ddagger }$ for AQP1 and AQP4 amounts to only 0.3 kcal/mol (Eq. (4)), thereby supporting the notion that $\Delta {G}^{{\rm o}}$ is small.

## Conclusion

Our above analysis of energetic contributions to single-file water transport underscores the importance of hydrogen bonds between the permeating water molecules and the channel wall. For most biological channels, intraluminal hydrogen bonds critically shape $\Delta {G}^{\ddagger }$. Channels facilitating very rapid water transport may constitute an exception. In the extreme case of GlpF, the energy required for water partitioning, $\Delta {G}^{{\rm o}}$, combined with the energy for H-bond-unrelated progression of the file contributes roughly 75% to $\Delta {G}^{\ddagger }$. In any case, the entropy difference between bulk water and intraluminal water appears to be negligible. This observation suggests that *E*_A_ may serve as a good approximation for $\Delta {G}^{\ddagger }$. The observation of an exceedingly large *E*_A_, i.e., an *E*_A_ out of proportion with the measured *p*_f_ value, indicates—in all likelihood—a non-porous water pathway.
